# Sensory Attributes, Microbial Activity, Fatty Acid Composition and Meat Quality Traits of Hanwoo Cattle Fed a Diet Supplemented with Stevioside and Organic Selenium

**DOI:** 10.3390/foods10010129

**Published:** 2021-01-08

**Authors:** Yong Geum Shin, Dhanushka Rathnayake, Hong Seok Mun, Muhammad Ammar Dilawar, Sreynak Pov, Chul Ju Yang

**Affiliations:** Animal Nutrition and Feed Science Laboratory, Department of Animal Science and Technology, Sunchon National University, Suncheon 57922, Korea; shin0048@naver.com (Y.G.S.); dhanus871@gmail.com (D.R.); mhs88828@nate.com (H.S.M.); ammar_dilawar@yahoo.com (M.A.D.); sreynakpov@gmail.com (S.P.)

**Keywords:** stevioside, organic selenium, Hanwoo cattle, fatty acid profile, oxidative status, sensory attributes

## Abstract

This study examined the effects of stevioside (S) and organic selenium (O-Se) supplementation on the sensory attributes, microbial activity, fatty acid composition, and meat quality traits of Hanwoo cattle (Korean native cattle). Twenty-four Hanwoo cattle (663 ± 22 kg body weight) were assigned to two dietary treatments for 8 months: control diet and 1% stevioside with 0.08% organic selenium supplemented diet. S and O-Se inclusion in the diet enhanced the final body weight, weight gain, and carcass crude protein (*p* < 0.05). Moreover, supplementation with S and O-Se had a significant effect on lowering the drip loss and shear force and enhanced the a* (redness) of the *longissimus dorsi* muscle (*p* < 0.05). The inclusion of dietary S and O-Se improved the sum of the polyunsaturated fatty acid (ΣPUFAs) content of the meat, and the oxidative status (TBARS) values during second week of storage decreased by 42% (*p* < 0.05). On the other hand, the microbial count tended to decrease (7.62 vs. 7.41 log_10_ CFU), but it was not significant (*p* > 0.05), and all sensory attributes were enhanced in the S and O-Se supplemented diet. Overall, these results suggest that supplementation of the ruminant diet with stevioside and organic selenium improves the growth performance, carcass traits, and meat quality with enriched PUFAs profile and retards the lipid oxidation during the storage period in beef.

## 1. Introduction

High meat quality characteristics of Hanwoo beef enhanced the consumer’s preference compared to the other imported beef, such as Holstein steer and Australian Angus [1]. Moreover, lower subcutaneous fat contents and higher ossification scores, marbling scores, and loin protein content were also observed in the Hanwoo carcasses compared to Australian Angus carcasses [2]. Since dietary management has an important role in the beef industry, the implementation of promising feeding strategies and supplementation with additives is an essential factor that can determine the physiochemical qualities of the meat. Natural and healthy feed additives have gained particular interest in the global livestock sector since the ban on antibiotic growth promoters (AGP) in animal feed [3].

Many beneficial organic bioactive molecules are derived from plants because of their normal metabolic reactions [4]. The mode of action of plant-based feed additives is increasing the digestibility and absorption by modulating the beneficial intestinal microbiota [5]. The Stevia genus comprises approximately 200 species of various herbs and shrubs [6]. *Stevia rebaudiana* Bertoni is an herbaceous perennial plant belonging to the *Asteraceae* family that is native to Paraguay but has also been cultivated in China, Taiwan, Korea, Malaysia, Canada, the United States, and some European countries. Stevioside (13-[2-O-beta-d-glucoprransyl-alpha-d-glucopy-ranosyl)oxy]kaur-16-en-18-oic acid-beta-D-glucopyranosyl) is the prominent steviol glycoside obtained from the leaves of *S. rebaudiana*; it is noncaloric and is stable at high temperatures over a wide range of pH [7]. Previous studies [8,9] reported that the stevioside exhibits numerous immunoregulation activities and has antibacterial properties, anti-inflammation properties, and maintenance of the blood lipid content. Previous studies showed that the stevia extract contains amino acids, polyphenols, and flavonoids and has antioxidant properties [10,11,12,13]. A few studies have been conducted on nonruminants, including pigs and poultry [14,15]. *S. rebaudiana* has been used as a sweetener and a feed additive because of the presence of various bioactive compounds.

A selenium deficiency has negative impacts on the health of animals and humans. Therefore, selenium is considered a vital component in diets. [16]. Selenium acts as an antioxidant against reactive oxygen species (ROS) through the glutathione peroxidase activity, a vital enzyme in the detoxification process [17,18]. Organic selenium is absorbed in the GI tract through active transmission and during the protein synthesis process and is deposited in tissues to fulfill the selenium requirements in organs and tissues [19]. In contrast, the utilization of organic selenium is more beneficial than inorganic selenium because of lower excretion via feces and urines. The antioxidant activity of selenium has been investigated in cattle [20,21], pigs [22,23], and poultry [24]. Nevertheless, no study has evaluated the combination of both stevioside (S) and organic selenium (O-Se) in the livestock sector.

This study examined the effects of S and O-Se as feed additives on the sensory attributes, fatty acid profiles, microbial activity, and meat quality traits of Hanwoo cattle.

## 2. Materials and Methods

### 2.1. Animal Ethics

The experimental protocol was approved by the Ministry for Agriculture, Forestry and Fishery in Korea, 2008 (SCNU-2017-1102).

### 2.2. Management of the Animals, Diets, and Experimental Design

A feeding trial was conducted for 8 months at the Animal Experimental Station, Sunchon National University, Suncheon, Korea. Briefly, 24 Korean native cattle (Hanwoo), aged 26 months and weighing approximately 663 ± 22 kg initial body weight, were enrolled in a completely randomized design. All animals were housed individually in raised cages maintained under environmentally controlled conditions, with an ambient temperature of 30 °C and average relative humidity of 70%. The cages were designed to enable the separate collection of feces and urine. At the end of the experimental period, all animals were slaughtered at the local abattoir to assess the carcass traits and meat quality parameters.

The animals were allotted randomly to two dietary treatments, with 12 animals per treatment group: control (basal diet) and treatment diet (basal diet + 1% stevioside, 0.08% organic selenium). A commercially available total mixed ration was used as the basal diet, and each Hanwoo animal was fed 12 kg per day. The treatment diet was prepared separately by incorporating 1% stevioside and 0.08% organic selenium on a weight: weight ratio basis. Table 1 lists the ingredients and chemical composition of the basal diets. The cattle were fed twice daily, divided into two feeding times (9:00 a.m. and 6:00 p.m.). The animals were given access to water ad libitum, and ventilation, lighting, and other management practices were implemented according to general practices. The CTC Bio Tech. Co. Ltd. Company, Seoul, Korea, provided the white-colored stevioside powder extracted from the *Stevia rebaudiana* leaves and organic Se. The purity of the extract was evaluated by high-performance liquid chromatography (HPLC) analysis of the stevioside sample and was determined to be 97%.

### 2.3. Growth Performances and Slaughtering

The body-weight gain was measured as the difference between the initial and final body weights. The initial body weight was measured at study commencement. The subsequent average weight gain (AWG) of Hanwoo cattle during the experimental period was determined using the live weight obtained monthly. The feed intake was measured daily. At the end of the experiment (8 months), all animals were slaughtered after 24 h of starvation. The animals were stunned, and the carcasses were exsanguinated and immediately eviscerated. Approximately 2.5 cm steaks of *Longissimus dorsi* muscle were obtained from the 13th rib. For each subsequent analysis, triplicate samples were obtained from each carcass after being stored at 4 °C for 24 h in a chilling room, as reported by Bostami et al. [25].

### 2.4. Proximate Composition, Carcass Traits, and Cholesterol Analysis

The proximate compositions of the muscles, fat, and connective tissues were determined by removing them manually and ground. An Ultra-Turrax homogenizer (IKA Werke, GMBH & Co. KG, Staufen, Germany) was used for the homogenization process. The moisture (930.15), crude protein (990.03), crude fat (991.36), and crude ash (942.05) compositions were evaluated according to the guidelines set up of AOAC [26].

The meat quality grade was scored as 1^++^, 1^+^, 1, 2, and 3, according to the Korean beef quality grading system [27]. The main evaluated parameters were the marbling score, meat color, fat color, texture, and maturity. The marbling score was determined on a 7-point scale (7 = abundant and 1 = trace). A 7-point scale was used for scoring meat color (7 = dark red and 1 = bright red), and fat color (7 = yellowish and 1 = creamy white). A scale of 1 to 3 was used to score the texture (1 = firm and 3 = soft) and maturity (3 = mature and 1 = youthful).

The cholesterol content of the *Longissimus dorsi* muscle was determined using the method described by King et al. [28]. Briefly, 5 g of the meat sample was saponified using a chloroform and methanol mixture (2:1 vol:vol) [29]. The saponified samples were analyzed by gas chromatography (GC, DS 6200, Donam Co., Seongnam, Gyeonggi-do, Korea) with a flame ionization detector and a Hewlett Packard HP-5 capillary column (J and W Scientific, Folsom, CA, USA) with a 0.32 internal diameter, 30 m length, and 0.25 µm polyethylene glycol-film thickness. The initial temperature of the setup was 250 °C for 2 min and was increased gradually to 290 °C at a rate of 15 °C/min. The final temperature was increased to 310 °C at 10 °C/min, and held at that temperature for 10 min. The other chromatographic conditions were as follows: injector and detector temperatures of 280 °C, split ratio of 50:1, and injected sample volume of 2 µL.

### 2.5. Meat Quality Analysis (Meat Color, Drip Loss, Cooking Loss, Water Holding Capacity (WHC), and Shear Force)

The meat color of the meat samples (in triplicate) was measured using a Chroma meter (Model CR-410, Konica Minolta Sensing Inc., Osaka, Japan). According to the Commission International de I’Eclairage (CIE) system, the color was classified by the CIE L* (lightness), CIE a* (redness), and CIE b* (yellowness) values. Cooking loss (in triplicate) is expressed as the percentage of weight loss and was evaluated by placing 1.5 cm-thick steaks of about 80 g in a polythene zipper bag, heating them in a water bath at 75 °C for 30 min, cooling them to room temperature, and holding them for 30 min. The cooked samples were cut (0.5 cm × 4.0 cm). The shear force was determined in each cooked meat sample using a Warner-Bratzler shear blade set (Lloyd Instruments Ltd., Hampshire, UK) by applying the following operating parameters: load cell of 50 kg, cross-head speed of 200 mm/min, and trigger force of 0.01 kgf. The drip loss (in triplicate) was determined as the weight loss during the suspension of a standardized sample (2 × 2 × 1 cm) sealed in a polythene bag at 4 °C after 7 days of storage. The water holding capacity (WHC) was determined using the method described by Grau et al. [30]. Briefly, 300 mg of the *Longissimus dorsi* muscle was placed in a filter-press device and compressed for approximately 2 min. The WHC was then calculated from triplicate meat samples, as a ratio of the meat film area to the total area using an area-line meter (Super PLANIX-a, Tamaya Technics Inc., Tokyo, Japan).

### 2.6. pH Value and Oxidative Stability of Meat

To determine the pH, approximately 5 g of *Longissimus dorsi* muscle (in triplicate) was cut into small pieces and homogenized with 45 mL distilled water for 60 s in an Ultra-Turrax (Janke and Kunkel, T25, Staufen Germany). Immediately after homogenization, the pH was measured using a digital pH meter (Docu-pH + meter, Sartorius, Columbus, OH, USA).

The thiobarbituric acid reactive substances (TBARS) were evaluated (in triplicate) using the procedure described by Witte et al. [31]. Briefly, 5 g of meat samples were mixed with 25 mL of 20% trichloroacetic acid (TCA) and homogenized for 30 s. Distilled water was added to prepare 50 mL of homogenate samples for centrifugation (3000× *g*, 4 °C, and 10 min). The supernatant was filtered through filter paper (Hyundai Co., Ltd., Seoul, Korea). Then, 5 mL of the filtrate was kept at room temperature for 15 h, and the absorbance was determined using a UV/VIS spectrophotometer (M2e, Molecular Devices, Sunnyvale, CA, USA). The TBARS value is expressed as micromoles of MDA/kg of meat.

### 2.7. Fatty Acid Analysis

The fatty acid composition of *longissimus dorsi* muscle samples (in triplicate) was evaluated using a direct method for the fatty acid methyl ester (FAME), as suggested by O’Fallon et al. [32] with a slight modification. Briefly, 1 g of minced meat sample was placed into a 15 mL Falcon tube and 0.7 mL of 10 N KOH in water and 6.3 mL of methanol were added. The tube was kept in a water bath (55 °C; 1.5 h), allowing proper permeation, hydrolyzation, and dissolution by vigorous shaking for 10 s every 30 min. After placing in a cold tap water bath, 0.58 mL of 24 N H_2_SO_4_ was added to precipitate K_2_SO_4_. The precipitated sample was placed again in a water bath (55 °C; 1.5 h) with strong shaking for 10 s every 30 min. After FAME synthesis, 3 mL of hexane was added and subjected to centrifugation for 5 min at 3000 rpm (Hanil, Combi-514R, Gimpo, Korea). The top hexane layer, which contained FAME, was dehydrated by passing through the anhydrous Na_2_SO_4_. The extracted and dehydrated hexane was placed into a GC vial and concentrated to 1.5 mL for analysis.

The fatty acid composition of the FAME was determined using an Agilent gas chromatography system (6890 N, Agilent Technologies, Santa Clara, CA, USA). Briefly, fat was extracted from minced meat using a chloroform-methanol (2:1 *v*/*v*) solution [28]. According to the AOAC [26] procedure, the prepared fatty acid methyl esters were dissolved in hexane before injection; 1 µL of the prepared sample was injected into the GC; the set-up injector temperature was maintained at 250 °C with a 100:1 split ratio, using a WCOT-fused silica capillary column (100 m × 0.25 m i.d., 0.20 µm film thickness; Varian Inc., Palo Alto, CA, USA) with helium flow. The oven conditions were 150 °C/1 min, 150–200 °C at 7 °C/min, 200 °C/5 min, 200–250 °C at 5 °C/min, and 250 °C/10 min. The setup detector temperature was 275 °C. Fatty acid peaks were evaluated using the retention time of fatty acid standards (47015-U, Sigma-Aldrich Corp., LLC., St. Lois, MO, USA). The proportion (%) against the total peak area was calculated from the peak area of each fatty acid identified.

### 2.8. Meat Microbial Analysis and Sensory Evaluation

Triplicates of *longissimus dorsi* muscle from each group were taken for the meat microbial analysis. A 25 g sample of meat was homogenized using 225 mL of a NaCl solution (0.85% *W*/*V*). Subsequently, 20 µL was obtained from 10-fold diluted solution and transferred into tryptic soy agar plates (Becton, Dickinson, and Company, Sparks, MD 21152, USA) using a sterilized triangle spreader for microbial enumeration. The colonies were counted immediately after incubation. The microbial number was determined as follows: number of colonies × 10 dilution value × (100/20) = multiplied value = log (multiplied value). The ultimate count was expressed as log_10_ CFU/g.

Ten samples from both treatment groups were examined by 10 members, all well-trained experts of the sensory panel at the Department of Animal Science and Technology, Sunchon National University, Suncheon, Korea. Moreover, individual testing booths and a controlled lighting facility were provided during the sensory evaluation process [33]. Each steak was cooked at approximately 150 °C and until the internal temperature reached 70 °C, which was determined by inserting a digital thermometer in the steak. Before evaluation by the panelists, the steaks were wrapped in an aluminum foil and kept under 65 °C in an oven. The flavor, tenderness, and juiciness were then evaluated by the panelists. A 7-point hedonic scale was used to express the value of each characteristic: 7 indicated desirable flavor, extremely tender, reddish color, high palatability, and good juiciness, while 1 was indicative of undesirable favor, extremely tough, pale color, low palatability, and extremely dry. The average value of the 10 panelists determined the color, flavor, tenderness, juiciness, and palatability of the cut.

### 2.9. Statistical Analysis

The data were analyzed using the General Linear Model (GLM) method of the Statistics Package Program (SAS, 2003, Version 9.1, SAS Institute, Cary, NC, USA). For the growth performance parameters, a group of two cattle served as the experimental unit. Individual animals served as the experimental unit for the carcass traits, the meat quality parameters, cholesterol content, fatty acid profile, pH, TBARS, microbial analysis, and sensory evaluation.

Y_ij_ = μ + α_i_ + e_ij_. (1)

where Y_ij_ is the response variable, μ is the general mean value, α_i_ is the effect of dietary supplementation, and e_ij_ is the random error. The mean values were compared using a student’s *t*-test. The level of significance considered for the tests was *p* < 0.05.

## 3. Results

### 3.1. Growth Performance, Proximate Analysis, and Cholesterol Content

Over the entire experimental period, the final body weight and body weight gain were significantly higher (*p* < 0.05) in the S and O-Se supplemented diet. On the other hand, the feed intake did not have a significant effect but was higher in the S- and O-Se-supplemented diet (Table 2).

Proximate analysis revealed a significantly higher crude protein content, lower crude fat (*p* < 0.05), and a numerically lower meat cholesterol amount due to S and O-Se supplementation even if it is not significantly different (Table 3).

### 3.2. Carcass Traits

Although the S- and O-Se-supplemented diet increases the carcass yield, back-fat thickness, and loin area, the changes were not significant (*p* > 0.05). Moreover, the changes in the meat quality were also not significant, including the marbling score, meat color, fat color, texture, and maturity (Table 4).

### 3.3. Meat Quality Analysis (Meat Color, Drip Loss, Cooking Loss, WHC, and Shear Force)

Considering the changes in the meat color, the redness (a*) was enhanced significantly in the S- and O-Se-supplemented diet (*p* < 0.05). On the other hand, no significant difference in surface lightness (L*) and yellowness (y*) was observed between the two treatments. Although S and O-Se addition did not affect the cooking loss and water holding capacity (WHC) (*p* > 0.05), the drip loss and shear force decreased significantly (*p* < 0.05) (Table 5).

### 3.4. pH Value and Oxidative Stability of Meat

**Figure 1:**
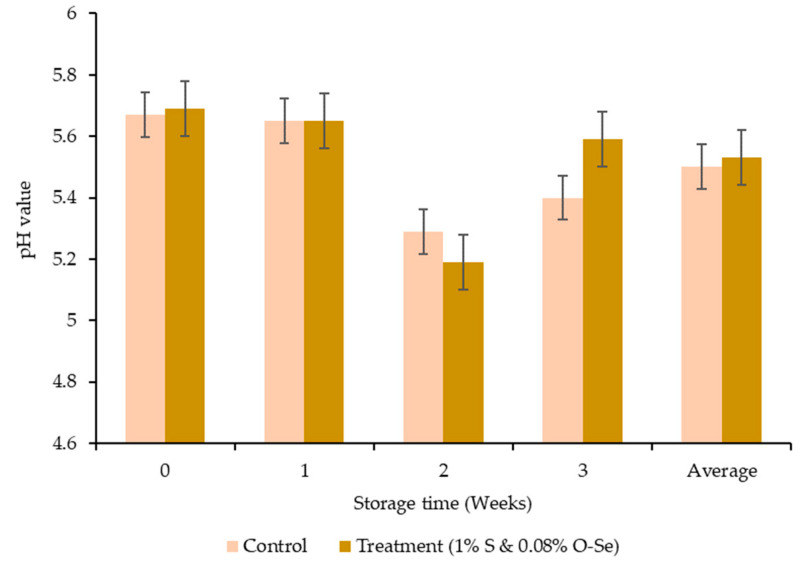
Effect of stevioside and organic selenium on the pH values in meats of Hanwoo cattle.

**Figure 2:**
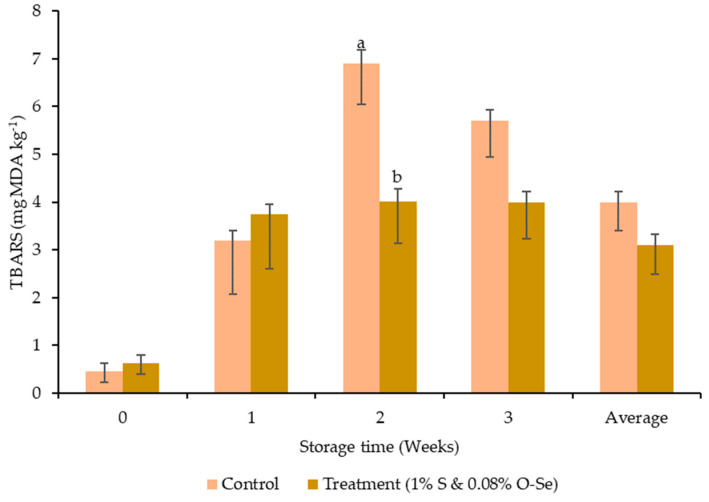
Effect of stevioside and organic selenium on the TBARS values in meats of Hanwoo cattle. Data presented as the mean ± s.e. bars at a specific time point with different letters show a significant difference (*p* < 0.05).

The pH in both treatments decreased gradually up to the second week of storage, with a subsequent increase at the end of the third week. On the other hand, no significant difference was observed between the control and S- and O-Se-added diet (Figure 1). The addition of S and O-Se resulted in significantly lower TBARS values during second week of storage (*p* < 0.05) (Figure 2).

### 3.5. Fatty Acid Analysis

Monounsaturated fatty acids (MUFA) had the highest proportion. They constituted more than 50% of the total fatty acid composition, followed by saturated fatty acids (SFA) and polyunsaturated fatty acids (PUFA), accounting for 37–39% and 0.6–0.9%, respectively. Oleic acid (C8:1, 47%) was the predominant fatty acid among MUFA, followed by palmitoleic acid (C16:1, 5%), while palmitic acid (C16:0, 24–26%, stearic acid (C18:0, 8%) and myristic acid (C14:0, 3%) were predominant among SFA. On the other hand, the decrease in SFAs content was not significant in the S- and O-Se-supplemented diet. Moreover, the nervonic monounsaturated fatty acid content and linoleic, di homo-gamma linolenic, and arachidonic PUFAs contents were also higher in the animals fed the S- and O-Se-supplemented diet (*p* < 0.05) (Table 6).

### 3.6. Microbial Analysis and Sensory Evaluation

**Figure 3:**
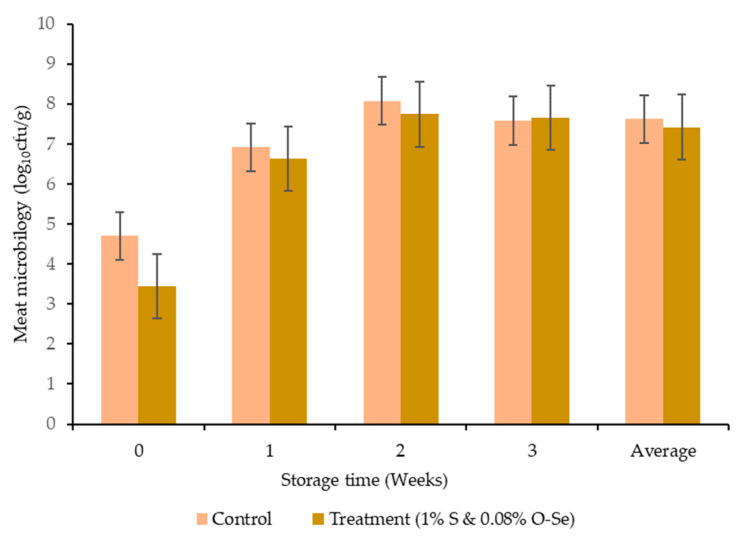
Effect of stevioside and organic selenium on the microbial content in meats of Hanwoo cattle.

The average meat microbial content was numerically lower, but not significant (7.62 vs. 7.41 log_10_ CFU) in the S- and O-Se-supplemented diet compared to the control treatment after the 3-week refrigerated storage period (Figure 3).

**Figure 4:**
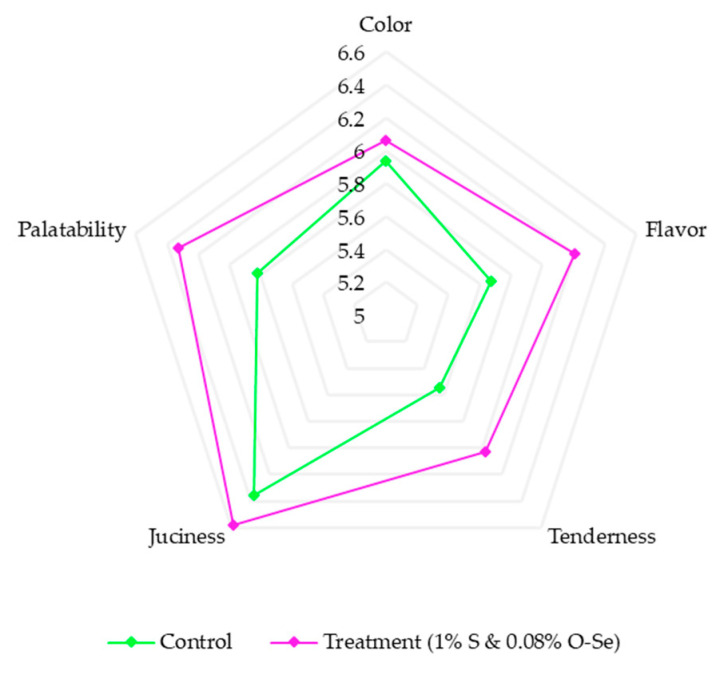
Effect of stevioside and organic selenium on meat sensory attributes of Hanwoo cattle.

Although no significant impact (*p* > 0.05) was observed for color, flavor, tenderness, juiciness, and palatability, the S- and O-Se-supplemented diet had a positive tendency on the sensory parameters (Figure 4).

## 4. Discussion

The incorporation of feed additives has gained popularity because of their benefits to human and animal health [34,35]. In particular, phytochemicals have biochemical properties, including antioxidant, antimicrobial, antistress, and nutrigenomic influences on the development of immunity and improved productivity [36,37]. Moreover, the incorporation of organic selenium can improve nutrient utilization by preventing the pro-oxidant effects on the gut system and has an antioxidant defense role against oxidative stress [38].

In the present study, 1% stevioside and 0.08% organic selenium supplementation significantly increased the weight gain and final body weight and enhanced the feed intake (FI). The palatability of the feed was enhanced because of the sweetness of the stevioside, and the animals increased their voluntary feed intake, resulting in increased body weight. In contrast, stevioside derivatives can stimulate the taste receptors (TASIR2/TASIR3) [39,40] and K^+^ channels in pancreatic β-cells [41], which can increase the stimulation of appetite and voluntary FI. Similar to the present findings, Han et al. 2019 [42] reported a linear increase in FI in goats given diets supplemented with 270 and 541 mg/kg stevioside. In contrast, Cho et al. [43] reported that the dietary intake of Hanwoo steers is not influenced by the inclusion of 65 mg/kg stevioside as an essential oil to the feed with 0.1% of diet supplemented rate. Furthermore, previous studies reported improved production parameters in pigs [44], layers [45], broilers [46], and cattle [47] owing to O-Se supplementation. The enhanced production performance may be associated with the developed antioxidant capacity and decrease in microbial pathogen colonization in the gut system because of the synergistic action of S and O-Se supplementation.

No studies have evaluated the effects of the dietary inclusion of S and O-Se on the carcass traits, meat quality, and sensory evaluation of cattle. A Se deficiency increases the susceptibility to various degenerative diseases in humans [48]. The incorporation of Se into animal feed has great potential to obtain Se-enriched meat that can alleviate Se deficiencies [49]. Dietary S and O-Se supplementation increased the moisture and crude protein content with a concomitant reduction in the crude fat content in meat taken from Hanwoo cattle. The lower crude fat content in meat might be associated with the lipolytic mechanism owing to the presence of polyphenols and flavonoids compounds in *S. rebaudiana* [50]. The increase in moisture content is caused by the inverse relationship between the meat fat and moisture content, which affects the meat tenderness and juiciness [51]. The cholesterol content was lower in the S and O-Se-included treatment, probably due to the cooperative mechanism of flavonoids in stevioside and glutathione peroxidase activity in organic Se [52], which can convert cholesterol to bile acids through the induced stimulation of enzymes activity. Consequently, it is catabolized and eliminated from the body.

Genetics and environmental factors, including the feeding strategies, affect the carcass quality traits directly [53]. Although no significant difference was observed in back-fat thickness of both treatments, Zhang et al. [54] stated that O-Se can provide some vitamins, amino acids, protein, and other nutrients for the body and can cause subsequent increase in fat accumulation. Similar to that, Choi et al. [55] also reported significant differences in the carcass length and back-fat thickness of pork meat obtained from animals fed stevia and charcoal-supplemented diets. Nevertheless, 0.3% stevia and charcoal inclusion did not significantly enhance the marbling, firmness, and color scores in pigs compared to the control treatment. Therefore, further investigations will be needed to determine the impact on the carcass traits based on different concentrations of stevioside and organic Se application.

The meat color and brightness are considered important visual factors that affect the consumer’s preference and purchasing decisions [56]. In the current study, the redness (a*) value was enhanced by the inclusion of S and O-Se in the cattle diet. The increase in meat redness may be associated with the reduced metmyoglobin (MMG) formation by antioxidants in both S and O-Se by delaying meat oxymyoglobin (OMG) oxidation. Choi et al. [55] also reported higher L*, a*, and b* values in pork meat supplemented with 0.3% stevioside and charcoal. The WHC was not significant in the two treatments, but the drip loss and shear force values were significantly lower in the S- and O-Se-supplemented group. The meat pH and lipid peroxidation affected meat drip loss [57]. Therefore, in the current study, the reduced lipid oxidation caused by S and O-Se supplementation may affect the lower drip loss value. Moreover, the S and O-Se supplemented diet had no adverse effect on the meat quality parameters.

High accumulated lactic acid content due to the metabolism of the glycogen reserves and releasing of H^+^ from ATP hydrolysis will lead lower muscle pH. Therefore, the carcass pH has a significant influence on the meat quality traits. During the 3 weeks in the S- and O-Se-included diet, the high pH of the meat probably affected the glutathione peroxidase enzyme activity, which can break down H_2_O_2_ to H_2_O and O_2_ [58] and facilitate an increase in meat pH. Moreover, in the current study, the TBARS value during the second week of the storage periods was 42% lower in the S- and O-Se-supplemented diet group than the control group. In addition to acting as a sweetener, previous studies reported the antioxidant properties of stevioside [59,60,61]. Moreover, plant polyphenolic compounds can prevent the oxidation activities in unsaturated fatty acids by scavenging free radicals [62] or singlet-oxygen quenching ability [63]. The glutathione peroxidase family (GPx) in organic Se can also delay the lipid oxidation reactions [64]. Overall, the synergistic effects of both S and O-Se reduced the average TBARS value owing to the presence of antioxidants.

The meat fatty acids composition depends on the lipogenesis activities in adipose tissues and the ruminal biohydrogenation process [65]. The dietary SFA has a direct correlation with cholesterol level and subsequent cardiovascular diseases. Nevertheless, PUFA positively exerts a lower cholesterol content and a significant reduction in human health risks [66]. In the present study, the PUFA content was significantly higher in the S- and O-Se-supplemented diet. This contrasts with a previous study [67], which noted a higher linoleic content in fattening steers fed a diet containing O-Se, possibly because of a reduction in biohydrogenation process and subsequent increase in the intestinal absorption of PUFA. A previous study [24] reported a lower MUFA and a higher PUFA proportion in the O-Se-supplemented group owing to the presence of antioxidants. Moreover, phytochemicals in plants indirectly performed their antioxidants activities against the depletion of PUFA from microbial biohydrogenation and enhanced the UFA concentration in the muscles [68]. Therefore, the combination of S and O-Se has a positive impact on the PUFA content in the meat.

A combination of microbes and endogenous enzymes leads to the deterioration of meat protein and consequently accelerates meat spoilage [69]. Synthetic preservatives are the main approach to inhibit microbial growth. On the other hand, preservatives need to be replaced by harmless natural compounds to avoid harmful effects on human health [70]. In the present study, the average microbial count tended to decrease in S and O-Se treatment during the refrigerated storage period, possibly because the GSH-Px enzymatic activity can eradicate harmful lipid peroxide and H_2_O_2_ from organisms, resulting in less favorable reproductive conditions for microbes. Hence, the microbial population tends to decrease gradually [54]. Moreover, antioxidant molecules in stevioside can scavenge free radicals that influence the retarded muscle oxidation process and reduce the microbial flora content in meat. In our study, the supplementation of S and O-Se did not modify the meat microbial count significantly. Hence, the incorporation of different concentrations of S and O-Se in cattle diets requires further study.

The sensory evaluation helps determine the consumer preference or acceptability of meat through an evaluation via sight, aroma, taste, and touch. In the present study, the supplementation of S and O-Se tend to positively influence color, flavor, tenderness, juiciness, and palatability of meat. Lipid peroxidation influences the deterioration of the above sensory properties of meat and the optimal quality of meat products [54,71]. Therefore, the antioxidant properties of S [72] and O-Se probably helps reduce the lipid oxidation process of meat and helps enhance the sensory properties. Interestingly, Zou et al. [70] reported that O-Se could retard the oxidation of OMG and lipids, resulting in a higher meat color score and meat stability. Furthermore, previous studies confirmed that the O-Se-incorporated diet in the animal feed improves the meat quality by preventing excessive dehydration and protects the meat sensory attributes [73]. Consistently, this study indicated that the synergism of S and O-Se in the cattle diet led to an improvement in the meat sensory attributes.

## 5. Conclusions

The addition of S and O-Se in the cattle diet enhanced the final body weight (FBW) and weight gain (WG). In addition, the carcass protein and moisture contents were improved, and the cholesterol content was reduced. Both S and O-Se inclusion improved the meat redness (a*) and reduced the meat drip loss and shear force value. The average TBARS value in S and O-Se supplemented diet during the second week was decreased by 42% and showed a higher meat ΣPUFA content than the control group. In this study, all sensory attributes tended to increase numerically and microbial flora contents also tended to decrease due to the S- and O-Se-incorporated diet. These results indicated that the supplementation of natural stevioside and organic selenium could be carried out to enhance the productive performance and the quality of meat in the livestock sector. Nevertheless, further studies will be needed to determine if different concentrations could provide more benefit in ruminant diets.

## Figures and Tables

**Table 1:** Feed ingredients and chemical composition of experimental diet for Hanwoo cattle.

Composition	Amount
Ingredient (%, as-fed basis)	
Corn grain	43.28
Corn gluten feed	11.81
Wheat	10.47
Palm kernel expeller	9.03
Coconut meal	8.29
Lupin	5.91
Tapioca	3.41
Molasses	3.05
Rapeseed meal	2.06
Wheat flour	1.97
Soybean meal	0.50
Vitamin mineral premix ^A^	0.22
Chemical composition (%DM)	
Total digestible nutrients (TDN)	74.12
Crude protein	12.50
Crude fat	3.61
Crude fiber	6.70
Crude ash	5.48
Calcium	0.84
Phosphorus	0.36
Neutral detergent fiber (NDF)	21.70
Acid detergent fiber (ADF)	10.70

^A^ Premix providing following nutrients per kg of diet: vitamin (Vit.) A, 9,000,000 IU; Vit. D_3_, 2,100,000 IU; Vit. E, 15,000 IU; Vit. K, 2000 mg; Vit. B_1,_ 1500 mg; Vit. B_2,_ 4000 mg; Vit. B_6,_ 3000 mg; Vit. B_12,_ 15 mg; Pan acid-Ca, 8500 mg; niacin, 20,000 mg; biotin, 110 mg; folic acid, 600 mg; Co, 300 mg; Cu, 3500 mg; Mn, 55,000 mg; Zn, 40,000 mg; I, 600 mg; Se, 130 mg.

**Table 2:** Effect of stevioside and organic selenium on growth performances of Hanwoo cattle (experimental period: 243 days).

Item	CON ^(1)^	TRT ^(2)^	SEM	*p*-Value
Initial BW	647.00	652.00	22.01	0.32
Final BW	717.79 ^b^	751.93 ^a^	31.47	0.04
Weight gain (kg)	0.30 ^b^	0.42 ^a^	0.14	0.03
Feed intake (kg/Day)	11.32	11.43	0.22	0.51

^a,b^ Means in the same row with different superscripts are significantly different; SEM = standard error of mean. ^(1)^ CON: control diet with no added stevioside and organic selenium; ^(2)^ TRT: treatment (basal diet + 1% stevioside + 0.08% organic selenium).

**Table 3:** Effect of stevioside and organic selenium on proximate analysis and cholesterol contents in the meats of Hanwoo cattle.

Item	CON ^(1)^	TRT ^(2)^	SEM	*p*-Value
Moisture (%)	56.83 ^b^	63.89 ^a^	1.23	0.01
Crude protein (%)	22.48 ^b^	25.26 ^a^	0.80	0.03
Crude fat (%)	16.62 ^a^	12.45 ^b^	0.95	0.01
Crude ash (%)	1.18	1.29	0.05	0.14
Cholesterol (mg/100 g)	50.27	49.74	0.04	0.92

^a,b^ Means in the same row with different superscripts are significantly different; SEM = standard error of mean; ^(1)^ CON: control diet with no added stevioside and organic selenium; ^(2)^ TRT: treatment (basal diet + 1% stevioside + 0.08% organic selenium).

**Table 4:** Effect of stevioside and organic selenium supplementation on carcass traits of Hanwoo cattle.

Items	CON ^(1)^	TRT ^(2)^	SEM	*p*-Value
Yield traits				
Carcass weight (kg)	446.68	449.57	20.46	0.87
Loin area (cm^2^)	100.50	103.33	4.42	0.67
Back fat thickness (mm)	16.67	18.33	2.81	0.68
Quality traits				
Marbling score ^(3)^	4.83	6.33	0.78	0.20
Meat color ^(4)^	4.83	4.83	0.17	1.00
Fat color ^(5)^	3.17	2.83	0.19	0.18
Texture ^(6)^	1.17	1.00	0.08	0.34
Maturity ^(7)^	2.00	1.89	0.16	0.21
Quality grade	0:3:1:2:0	2:1:3:0:0	–	–
Meat point ^(8)^	3.17	3.83	0.40	0.26

SEM = standard error of mean; ^(1)^ CON: control diet with no added stevioside and organic selenium; ^(2)^ TRT: treatment (basal diet + 1% stevioside + 0.08% organic selenium); ^(3)^ Marbling score: 7 = abundant, 1 = trace; ^(4)^ meat color: 1 = bright red and 7 = dark red; ^(5)^ fat color: 1 = creamy white and 7 = yellowish.; ^(6)^ texture: 1 = firm and 3 = soft.; ^(7)^ maturity: 1 = young and 3 = youthful; ^(8)^ meat point: 1++: 5 point, 1+: 4 Point, 1: 3 Point, 2: 2 Point, and 3: 1 Point.

**Table 5:** Effect of stevioside and organic selenium on meat quality of *Longissimus dorsi* muscle from Hanwoo cattle.

Item	CON ^(1)^	TRT ^(2)^	SEM	*p*-Value
Meat color				
CIE L*	29.04	29.21	0.73	0.87
CIE a*	14.94 ^b^	18.45 ^a^	0.81	0.03
CIE b*	3.58	5.87	0.70	0.07
Drip loss (%)	21.14 ^a^	16.48 ^b^	0.75	0.00
Cooking loss (%)	15.81	16.30	0.71	0.70
WHC (%)	12.81	13.30	1.26	0.78
Shear force (kg)	5.38 ^a^	3.93 ^b^	0.35	0.01

^a,b^ Means in the same row with different superscripts are significantly different; SEM = standard error of mean; ^(1)^ CON: control diet with no added stevioside and organic selenium; ^(2)^ TRT: treatment (basal diet + 1% stevioside + 0.08% organic selenium).

**Table 6:** Fatty acid profile of *Longissimus dorsi* muscle from Hanwoo cattle fed diets containing stevioside and organic selenium.

Items	CON ^(1)^	TRT ^(2)^	SEM	*p*-Value
C10:0	0.03	0.04	0.00	0.08
C12:0	0.08	0.08	0.00	0.69
C14:0	3.35	3.39	0.11	0.80
C15:0	0.20 ^b^	0.25 ^a^	0.01	0.008
C16:0	26.65 ^a^	24.05 ^b^	0.60	0.04
C17:0	0.92 ^a^	0.15 ^b^	0.03	<0.0001
C18:0	8.33	8.67	0.15	0.13
C20:0	0.08 ^a^	0.07 ^b^	0.00	0.004
C14:1	0.30 ^a^	0.25 ^b^	0.02	0.04
C16:1	5.92 ^a^	5.62 ^b^	0.06	0.007
C18:1	47.19	46.09	0.58	0.24
C24:1	0.06 ^b^	0.08 ^a^	0.00	0.01
C18:2	0.06 ^b^	0.10 ^a^	0.01	0.004
C18:3	0.22 ^b^	0.36 ^a^	0.04	0.008
C20:3	0.13 ^b^	0.16 ^a^	0.01	0.04
C20:4	0.20 ^b^	0.26 ^a^	0.01	0.03
ΣSFA ^(3)^	39.69	36.75	0.72	0.16
ΣMUFA ^(4)^	53.46	52.04	0.58	0.13
ΣPUFA ^(5)^	0.63 ^b^	0.88 ^a^	0.05	0.04
ΣPUFA/SFA	0.015	0.024	0.04	0.14

^a,b^ Means in the same row with different superscripts are significantly different; SEM = standard error of mean; ^(1)^ CON: control diet with no added stevioside and organic selenium; ^(2)^ TRT: treatment (basal diet + 1% stevioside supplementation + 0.08% organic selenium); ^(3)^ SFA: saturated fatty acid; ^(4)^ MUFA: monounsaturated fatty acid; ^(5)^ PUFA: polyunsaturated fatty acid.

## Data Availability

The data presented in this study are available in the article.
